# Impact of COVID-19 on asthma control in Kirkuk City: an analysis of post-pandemic trends

**DOI:** 10.25122/jml-2023-0175

**Published:** 2024-03

**Authors:** Bilal Jamal Kamal, Mohammed Ali Khalaf

**Affiliations:** 1 Department of Medicine, College of Medicine, University of Kirkuk, Kirkuk, Iraq

**Keywords:** Asthma, COVID-19, post-COVID-19 complications

## Abstract

Bronchial asthma, a common chronic respiratory disease, should be managed and controlled correctly to prevent symptoms and maintain a good quality of life. Viral upper respiratory infections, especially the widespread COVID-19 virus, can exacerbate asthma. This study investigated the impact of COVID-19 severity (mild, moderate, severe) on asthma control compared to a control group without COVID-19. Asthma control was assessed using Asthma Control Test (ACT) scores and spirometry before and after COVID-19 infection. Statistical analysis revealed a significant decline (*P* = 0.001) in asthma control following mild to moderate COVID-19 recovery, evidenced by increased asthma symptoms, lower ACT scores, difficulty managing asthma, and increased need for asthma medication.

## INTRODUCTION

The COVID-19 pandemic has significantly impacted global health, with millions infected worldwide. As a respiratory illness, COVID-19 raises concerns for individuals with pre-existing respiratory conditions. Asthma is a chronic inflammatory illness of the airways, characterized by frequent exacerbation of wheezing, dyspnea, chest tightness, and coughing. Understanding how COVID-19 affects asthma control and symptoms will help healthcare practitioners treat patients with asthma [1,2].

Given that respiratory infections can worsen asthma control, the potential interaction between COVID-19 and asthma has received interest. Patients with asthma may be more susceptible to severe respiratory illnesses caused by viruses. Studies suggest that COVID-19 can exacerbate asthma symptoms, cause exacerbations, and complicate overall asthma management. The complex relationship between COVID-19 and asthma involves the direct effects of the virus on the respiratory system, its ability to induce systemic inflammation, and potential disruptions to asthma treatment techniques [3-5].

Understanding how COVID-19 affects asthma management is important for various reasons. First, it can identify patients with asthma at high risk of severe COVID-19 outcomes, leading to targeted prevention and therapeutic care. Second, it might reveal how COVID-19 exacerbates asthma, potentially leading to improved management strategies. Finally, assessing the influence of COVID-19 on asthma control can inform pandemic-related public health initiatives and policies. Understanding these effects will ultimately improve the treatment and well-being of patients with asthma [4-6].

Even after a mild-to-moderate COVID-19 infection, long-term consequences can impact the gastrointestinal, endocrine, central neurological, and respiratory systems [7]. While vaccines and antivirals help mitigate COVID-19 symptoms, research suggests that type 2 (non-atopic) asthma may be associated with a higher risk of severe COVID-19 complications, although the specific reasons behind this association remain unclear. COVID-19 has been shown to affect asthma control since the outbreak began. Studies investigating the effect of COVID-19 on the respiratory health of patients with asthma have shown conflicting results [8]. For example, a Hong Kong study found that patients with asthma who recovered from mild-to-moderate COVID-19 reported worse asthma control, lower Asthma Control Test (ACT) scores, and increased reliance on intensive asthma medication. Similarly, a Portuguese study observed a significant decrease in ACT scores following COVID-19 infection [7,8]. Studies show that mild-to-moderate COVID-19 can aggravate asthma symptoms, reduce ACT scores, and increase the requirement for asthma maintenance treatment. However, studies involving children have shown no significant impact of COVID-19 on asthma control. This highlights the need for continued research across different populations. Healthcare professionals must stay up-to-date on the latest research to ensure optimal management of asthma and COVID-19. This study aimed to contribute to the ongoing research by examining the impact of COVID-19 infection on asthma control in adult patients with asthma [9,10].

## MATERIAL AND METHODS

### Study design and participants

This study included 500 patients diagnosed with asthma (>18 years old) recruited from Azadi Teaching Hospital in Kirkuk City and various private clinics between 1st October 2021 and 1st January 2023. All patients had pre-existing asthma and were being treated with inhalers. Within the 30 to 270 days preceding their clinical examination, 250 had complained of mild to moderate COVID-19 infection, whereas the other 250 had no history of COVID-19 infection.

**Inclusion criteria:** Adult patients with asthma (≥18 years old) residing in Kirkuk City with documented mild-to-moderate COVID-19 infection at least 5 months prior to enrollment and regular follow-up in an asthma clinic or outpatient department were recruited.

**Exclusion criteria:** Patients visiting the emergency department or the outpatient clinic at Azadi Teaching Hospital for COVID-19 complications within 5 months of enrollment were excluded to minimize selection bias.

### Data collection

In this study, mild cases of COVID-19 were defined as those presenting typical symptoms such as cough, fever, malaise, sore throat, myalgia, headache, nausea, loss of smell and taste, vomiting, and diarrhea, but without shortness of breath, dyspnea, or abnormal findings in chest imaging. Patients with symptoms of lower respiratory disease, as determined by clinical examination or imaging, and oxygen saturation (SpO2) of 94% or above, as evaluated by pulse oximetry, were classified as having moderate SARS-CoV-2 disease.

Clinical data, including asthma medication, ACT scores, spirometry results, comorbidities, date of COVID-19 infection, hospitalization, COVID-19 complications, and vaccination status, were collected from the patient's clinical records at the time of their hospital admission. Demographic information such as gender, age, and smoking status were also recorded. The levels of asthma control were assessed using ACT scores, ranging from 5 to 25, where higher scores indicated better asthma control. The results were compared with the patient’s previous readings before COVID-19 infection. A difference of three points in ACT score was considered a minimum important difference (MID). Asthma was categorized as controlled if the ACT score was 20 or higher, partially controlled if the score ranged between 16 and 19, and uncontrolled if the score was 15 or lower. Lung function was assessed at the respiratory clinic from Azadi Teaching Hospital using a Spirobank II Smart instrument with disposable turbines. Spirometry data was analyzed according to updated spirometric reference values.

### Statistical analysis

The study utilized SPSS Statistics 26 for data analysis. Descriptive statistics were used to summarize participant characteristics (both clinical and demographic) using either mean and standard deviation (SD) for normally distributed data or median with interquartile range (IQR). Group comparisons between patients with asthma and prior COVID-19 infection and those without COVID-19 were conducted using Chi-squared or Fisher's exact tests (based on data type and sample size).

## RESULTS

The study included 500 patients with asthma, with 250 patients having a history of mild to moderate COVID-19 infection and 250 patients without a history of COVID-19. The mean baseline forced expiratory volume in 1 second (FEV1) and forced vital capacity (FVC) were 2.08 ± 0.75 and 3.18 ± 98, respectively, and the mean baseline FEV1 to FVC ratio was 66.6 ± 5.8 (Table 1). The mean ACT at the immediate prior visit was 20.0 ± 2.4, and at the enrolment visit, 18.4 ± 4.3 (good control). 190 (38%) of patients had a history of exacerbations 12 months before enrolment. In addition, 9% (45 patients) reported a decline in their asthma control that required an increase in their asthma maintenance medication by at least one GINA step (95% CI, 1.122–10.734; *P* < 0.001). A significantly greater proportion of patients with prior COVID-19 infection (22%, *n* = 55) had a decline of more than 3 points in their ACT score compared to the control group (4%, *n* = 10) (*P* < 0.001). Odds ratio (OR) analysis showed that patients with prior COVID-19 were 5.878 times more likely to experience a reduction of >3 points in their ACT score (95% CI, 2.364-8.837; *P* < 0.001). In addition, the OR for uncontrolled asthma in the COVID-19 group was 2.875 (95% CI, 1.145–7.348; *P* < 0.001). Furthermore, patients with prior COVID-19 infection demonstrated an 8.659 times greater likelihood of transitioning from controlled to uncontrolled asthma compared to the control group (95% CI, 3.764-23.847; *P* < 0.001). The results are summarized in Table 2.

**Table 1:** General characteristics of participants

Characteristics	COVID-19	Control	Total	*P* value
Age (years)	46 ± 10.2	44.6 ± 6.4	45.3 ± 9.2	0.08
Age of asthma onset (years)	19.8	20.23	20.14	0.05
Male	182 (73%)	128 (51%)	310 (62%)	
Female	93 (37%)	97 (39%)	190 (38%)	
Exacerbation required medical attention in the previous 12 months	40 (16%)	45 (18%)	85 (34%)	0.2
COVID-19 vaccine completion (more than two doses for more than 14 days)	225 (90%)	225 (90%)	450 (90%)	0.001
Baseline FEV1 (L)	2.12 ± 0.82	2.05 ± 0.7	2.08 ±0.75	0.3
FEV1 baseline (percent predicted)	89.2 ± 23.5	91.8 ± 12.4	90.11 ± 34	0.5
Baseline FVC (L)	3.12 ± 1.3	3.23 ± 0.67	3.18 ± 98	0.8
Baseline FVC (percent predicted)	104.5 ± 12.3	117.4 ± 66.3	11.9 ± 39.2	0.3
Baseline FEV1/FVC	65.8 ± 26.9	67.4 ± 84.7	66.6 ± 5.8	0.6
**ACT** score at the previous visit	20.0 ± 1.2	20.0 ± 4.7	20.0 ± 2.4	0.7
**ACT** score at enrollment	16.8 ± 3.9	20.0 ± 3.2	18.4 ± 4.3	<0.0001
**Asthma control at the most recent visit based on ACT score**				
Controlled	130 (52%)	154 (58%)	284 (56.8%)	
Partially controlled	75 (30%)	85 (34%)	160 (32%)	
Uncontrolled	45 (18%)	11 (8%)	56 (11%)	
Asthma control at enrollment based on ACT score	95 (38%)	120 (48%)	215 (43%)	
Partially controlled	50 (10%)	110 (22%)	160 (32%)	
Uncontrolled	115 (28%)	10 (2%)	125 (25%)	
Escalation of asthma treatment	205 (41%)	125 (25%)	330 (66%)	

**Table 2:** Odds ratios for changes in asthma control

	OR	95% CI	*P* value
≥3 ACT points.	5.878	2.364–8.837	0.016
Increased asthma medication (1 GINA step)	3.918	1.122–10.734	0.017
Uncontrolled asthma	2.875	1.145–7.348	0.006
Developed uncontrolled asthma	8.659	3.764–23.847	0.004

## DISCUSSION

COVID-19 infection directly damages the lungs, specifically the airways and air sacs (alveoli). This damage can worsen chronic respiratory conditions like asthma or chronic obstructive pulmonary disease (COPD) [11,12]. This study investigated the impact of prior mild-to-moderate COVID-19 infection on asthma control in adults already diagnosed with asthma. We recruited 500 participants with an average age of 45.3 years. The group consisted of 62% male and 38% female participants. Furthermore, 89% of the participants had completed the COVID-19 vaccine. Our findings align with the study of Najim *et al*. [13] in Kirkuk, who found that most patients affected were older, with males being more commonly affected. Similarly, another study in Baghdad reported that most patients with COVID-19 were above 50 years old, and men were more likely to be affected [14,15].

The findings of this study suggest an intermediate effect of COVID-19 infection on asthma management since all patients had been out of quarantine for at least 14 days and had moderate COVID-19. COVID-19 can cause respiratory symptoms such as cough and dyspnea, and it can worsen acute asthma, making it more challenging to control symptoms during the acute illness. However, what is particularly intriguing are the consequences that persist after COVID-19 infection, referred to as long-COVID syndrome or post-COVID syndrome. This condition involves persistent neuropsychiatric and physical symptoms extending beyond 12 weeks post-infection [16,17].

As COVID-19 has the potential to affect any system in the body, patients with pre-existing chronic respiratory disorders like asthma or COPD are at increased risk of experiencing various respiratory complications, ranging from moderate to severe. Therefore, it is reasonable to assume that individuals with severe, uncontrolled asthma may also be more susceptible to contracting COVID-19 and experiencing its associated complications [18,19]. The study found a decline in asthma control over time, as indicated by a decrease in the mean ACT score from 20.0 ± 2.4 at the immediate prior visit to 18.4 ± 4.3 at the enrollment visit. Several other studies also support this finding, suggesting that a significant proportion of patients with asthma previously infected with SARS-CoV-2 experienced worsening asthma symptoms during the infection period [13,14]. On the other hand, it was discovered that the prevalence of severe COVID-19 in patients with asthma was not any higher than in non-asthmatics, particularly when compared with patients with other comorbidities such as hypertension and diabetes [19,20].

Studies show that well-controlled asthma, through proper treatment and follow-up, reduces the risk of severe COVID-19 complications. For instance, Aveyard *et al*. [17] reported a lower risk of severe COVID-19, particularly in asthmatics using inhaled corticosteroids (ICS) who contracted the virus. However, our study revealed a decline in asthma control following recovery from a mild to moderate COVID-19 infection. This deterioration was evident in various areas, such as an increase in the dosage of asthma maintenance medication, a drop in the ACT score, and a higher proportion of patients with poorly managed asthma during follow-up. Notably, 9% of the patients complained of worsening asthma control, and the odds ratio (95% CI, 1.122–10.734; *P* < 0.001) indicates a significant association between COVID-19 infection and the increased risk of requiring intensified treatment to effectively manage asthma symptoms [20]. The decline in asthma control is subjective, as reflected by changes in the ACT score, but it may also be evident in the need to escalate asthma maintenance medication [13]. This aligns with findings from a Norwegian study on mixed hospitalized and home-isolated COVID-19 patients, where those with chronic respiratory diseases like asthma experienced persistent symptoms even six months after the initial infection [14,19]. The connection between asthma and the long-term consequences following COVID-19 infection discovered in this study may be linked to the pathophysiology of the TH2 pathway, suggesting a flare-up of the autoimmune system. Another hypothesis suggests that neuroinflammation, viral neurotropism, and neuroimmunomodulation via the vagal nerves, processes involved in SARS-CoV-2 infection, could contribute to a state of cough hypersensitivity, worsening asthma control after COVID-19 [21,22,23].

### Limitations

This study has limitations. First, recruiting patients from a single asthma clinic might have introduced selection bias, potentially excluding those who received care elsewhere or lacked regular follow-up. This could limit the generalizability of the findings to the broader asthma population. Second, the study lacks a control group of patients with asthma who did not have a history of COVID-19. Without a control group, it becomes challenging to establish a direct causal relationship between mild-to-moderate COVID-19 infection and the subsequent control of asthma symptoms.

## CONCLUSION

Despite experiencing only mild-to-moderate COVID-19 infection, patients with asthma in our study reported worsening asthma symptoms, as evidenced by lower ACT scores and increased need for escalated medication regimens, indicating poorly controlled asthma following recovery.
